# Selective Laser Melting (SLM) Additively Manufactured CoCrFeNiMn High-Entropy Alloy: Process Optimization, Microscale Mechanical Mechanism, and High-Cycle Fatigue Behavior

**DOI:** 10.3390/ma15238560

**Published:** 2022-12-01

**Authors:** Jianrui Zhang, Yabin Yan, Bo Li

**Affiliations:** 1 School of Mechanical and Power Engineering, East China University of Science and Technology, Shanghai 200237, China; 2 Shanghai Collaborative Innovation Center for High-End Equipment Reliability, Shanghai 200237, China

**Keywords:** selective laser melting, high-entropy alloys, CoCrFeNiMn, process optimization, microscale mechanical properties, fatigue, nano-twins

## Abstract

The equiatomic CoCrFeNiMn high-entropy alloy (HEA) possesses excellent properties including exceptional strength–ductility synergy, high corrosion resistance, and good thermal stability. Selective laser melting (SLM) additive manufacturing facilitates the convenient fabrication of the CoCrFeNiMn HEA parts with complex geometries. Here, the SLM process optimization was conducted to achieve a high relative density of as-built CoCrFeNiMn HEA bulks. The mechanisms of process-induced defects and process control were elucidated. The microscale mechanical behaviors were analyzed through in situ scanning electron microscopy observation during the compression tests on micro-pillars of the as-built HEA. The stress–strain characteristics by repeated slip and mechanism of “dislocation avalanche” during the compression of micro-pillars were discussed. The high-cycle fatigue tests of the as-built HEA were performed. It was found that a large number of nano-twins were induced by the fatigue, causing a non-negligible cycle softening phenomenon. The effects of promoted ductility due to the fatigue-induced nano-twins were illustrated. This work has some significance for the engineering application of the SLM additively manufactured CoCrFeNiMn HEA parts.

## 1. Introduction

The concept of multi-component high-entropy alloys (HEAs) was first proposed by Yeh et al. [1]. The number of elements comprising HEAs ranges from five to thirteen, and their molar ratios are all greater than 5% but less than 35% [2] By selecting appropriate constituent elements and adjusting their ratios, HEAs exhibit significant mechanical properties at high temperatures, good strength, ductility, and fracture toughness, as well as superparamagnetism, superconductivity, and excellent radiation resistance at low temperatures [3]. Among them, Professor Cantor discovered CoCrFeNiMn, a typical representative of HEAs, in the late 1970s and early 1980s. This alloy has excellent mechanical properties, surpassing high-strength steel and high-temperature nickel-based alloys [4]. However, compared with traditional alloy materials, HEAs have complicated compositions, and the research on material design and manufacturing is a long-lasting process. The pathway to improve the manufacturing capacity is a key to promoting its development.

Many scholars have studied HEAs [5,6,7], but most focus on design principles, metallurgical mechanisms, phase changes, micro-structures, and properties of HEAs manufactured by conventional technologies. Several AM methods have been successfully implemented to fabricate structures with HEAs [8]. The powder bed fusion (PBF) processes, specifically the selected laser melting (SLM) technique, have been widely adopted [9]. SLM is a powder-bed additive manufacturing technology, which is also advantageous for producing complex-shaped parts [10]. During the SLM process, rapid melting and solidification are attributable to short-time interaction between laser energy and metal powder, which facilitates the formation of fine microstructure and improves the mechanical properties of the parts [11]. Meanwhile, ultra-fast cooling helps prevent the formation of intermetallic compounds and element dissolution. Thus, SLM was recently also used for HEA manufacturing, and some studies on the manufacturing, microstructure, and tensile properties of these alloys have been reported [12]. However, rapid solidification results in a large temperature gradient, even residual stress, and cracks in the parts. 

The effect of SLM process parameters on the quality of the parts has been studied in a large body of literature, including mild steels [13], iron-based materials [14], nickel-chromium alloys [15], and high-temperature nickel-based alloys [16]. It has been highlighted that even small changes in processing parameters have a significant effect on the physical properties and microstructure of the final parts [17], and even give rise to major defects in the parts, e.g., unreasonable process parameters [18], internal defects induced by incomplete laser scanning filling [19], geometric defects and macroscopic deformations under residual stress generated by apparent thermal gradients in the manufacturing process [20], severe notching or deformation of the support bar. The literature [21] illustrates the causes of these defects and concludes that the mechanical and biological properties of the parts are seriously affected. It is also found in the literature [22] that the fatigue life of SLMed parts under multi-axle load is significantly reduced due to stress concentration caused by internal defects compared to forgings and that defects also affect the manufacturability, dimensional accuracy and surface roughness of SLM lattice structure [23]. To eliminate or reduce the defects resulting from the SLM manufacturing technology, a large number of scholars have conducted experimental research and revealed the effect of process parameters on the quality and mechanical response of the parts to find the best SLM process parameters and eliminate the mismatches between design and manufacturing [24]. Some scholars also hope to optimize defects by post-processing, such as heat treatment [25] and chemical methods [26]. However, only manufacturing defects can be reduced in various ways, and it is difficult to entirely eliminate all the geometric defects caused by the additive manufacturing process [27].

Nearly 130 factors affect the quality of the final parts [28], among which there are several key control parameters: laser power P (W), scanning speed ν (mm·s^−1^), laser spot diameter D (mm), powder layer thickness h (μm), scanning spacing H (μm), etc. In addition to emphasizing the effect of single factors on the density of SLMed parts, more scholars argue that stable part quality can be guaranteed after carefully considering all the parameters and their interactions. To comprehensively study the effect of different parameters on SLM, the concept of energy density [29] is introduced, expressed by ψ (J/mm^3^). The most common energy density equation is as follows:

(1) $$\psi =\frac{p}{\nu hd}$$

where ν is the scanning speed, p is the laser power, *d* is the layer thickness, and h is the scanning spacing.

Several AM methods have been successfully implemented to fabricate structures with HEAs. Some scholars have developed and manufactured HEA parts by SLM [30]. For example, Brif et al. [31] have manufactured high-strength and high-ductility CoCrFeNi HEA products by SLM. We can analyze single-track, single-layer, and multi-layer separately to study how to manufacture HEAs by SLM. Piglione et al. [32] have manufactured CoCrFeMnNi HEAs by SLM and found that the direction of grain growth was consistent with the direction of maximum heat flow during single-track forming; during multi-layer manufacturing, the solidified molten pool was remelted in adjacent tracks or continuous layers for laser melting, and the competitive growth optimized the grain size. Johnson et al. [33] have proposed a prediction criterion based on the geometric size of the molten pool to predict the manufacture of CoCrFeMnNi HEAs. Specifically, the process parameters are defined by the thresholds of L/W, W/D, and D/t, where L, W, and D correspond to the length, width, and depth of the molten pool, respectively, and t corresponds to the length and width of the molten pool and thickness of the deep powder layer.

Generally, the SLM has made significant progress in the past 20 years. However, there is still a significant gap in developing into a mature technology, especially in productivity, quality control, and repeatability, which poses severe challenges for critical components [34]. Moreover, the vast majority of studies on SLM focus on a few alloys, e.g., titanium alloys [35], nickel-based alloys [36], and stainless steels [37]. Further research on the SLM process, forming characteristics, and mechanical properties of high-entropy materials can further improve the properties of Cantor HEAs [38] and expand their application scope.

In this research, by investigating the SLM process of Cantor HEAs, the density of the parts can reach up to 98.87%, but the density of the parts will slightly decrease when the laser energy density continues to increase, and finally stays at about 97%. This research also finds that laser rapid non-equilibrium melting and solidification lead to that a large number of nano twin crystals induced by fatigue stress existing near high-density low-angle grain boundaries and micro-plastic deformation and ductility are improved in the low-load high-cycle fatigue process and provides a theoretical basis for controlling SLM forming quality of Cantor HEAs.

## 2. Experimental Method and Material

### 2.1. SLM Details

A professional SLM device is used in the experiment, including a laser beam system, mechanical motion system, control system, scanning system, material delivery system, and atmosphere protection system. Its parameters are presented in Table 1. Since the powder is completely melted throughout the process, it is essential to protect the machined parts from oxidation, with no more than 0.2% oxygen in an argon atmosphere throughout the manufacturing process.

**Figure 1:**
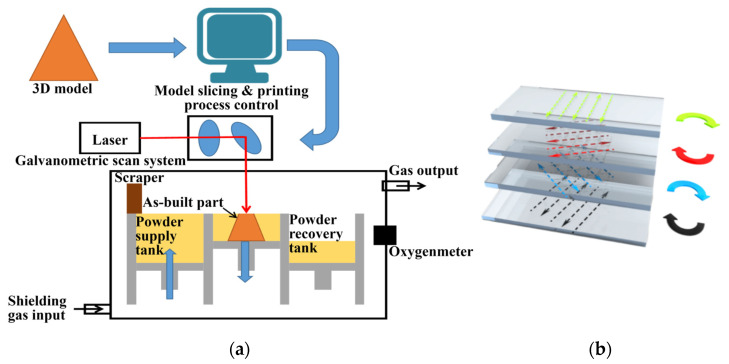
Schematic diagrams of SLM device (**a**) and laser scanning strategy (**b**).

In this research, the CoCrFeNiMn spherical powder produced by gas atomization is provided by Jiangsu Vilory Advanced Materials Technology Co., Ltd. (Suzhou, China), and its size is D_10_ = 24.6 μm, D_50_ = 36.2 μm and D_90_ = 55.8 μm, respectively. The scanning strategies of this paper are based on the recommendations of the literature [39]. The filling scanning strategy is chosen, i.e., interlaced scanning: after each layer is scanned, the next layer is canned after rotating 67°, and the specific scanning strategy is shown in Figure 1, by which the risk of unmelting between adjacent tracks can be minimized.

### 2.2. Measurement of Density

The density can be measured in many ways. By comparing the advantages and disadvantages of the Archimedean method, helium pycnometer method, and cross-sectional micrograph method in measuring the density of SLMed parts, the literature [40] concludes that: when the helium pycnometer method is adopted, the mass and volume of specimens should be measured separately, as the most accurate measurement method, but this method is relatively cumbersome and less efficient. In contrast, the results of the Archimedean method are very similar to those of the helium pycnometer method, but the efficiency is greatly improved. Therefore, the Archimedean method is a reliable estimation method in a deionized water environment.

**Figure 2:**
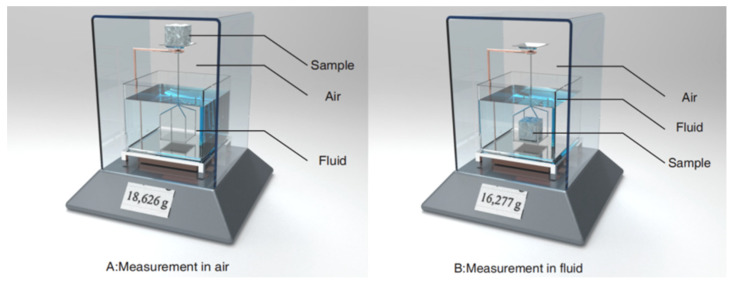
Measurement in air and liquid.

The mass of parts is measured by analytical balance with an accuracy of 0.1 mg. As shown in Figure 2, the sample is weighed separately in air and liquid. The literature [41] shows that powder dried at a high temperature can significantly reduce the porosity of the parts (≤35%). In this research, the powder has been dried at a high temperature (200 °C, 12 h), of which the effect will not be further discussed in this research. In addition, each sample is measured 10 times, and the samples are dried (110 °C, 30 min) before measurement to fully evaporate the water from the pores to not affect the air quality in the subsequent measurement. According to the literature [40], the density of the parts is measured by the Archimedean method in softened water, and the following measures are taken before measurement: ① cleaning the samples and removing the powder stuck on the surface of the parts using ultrasound; ② fully drying the samples between measurements, immersing them in a beaker containing softened water and gently brushing before each immersion measurement to ensure that all visible air bubbles are expelled.

### 2.3. High-Cycle Fatigue Experiment Details

**Figure 3:**
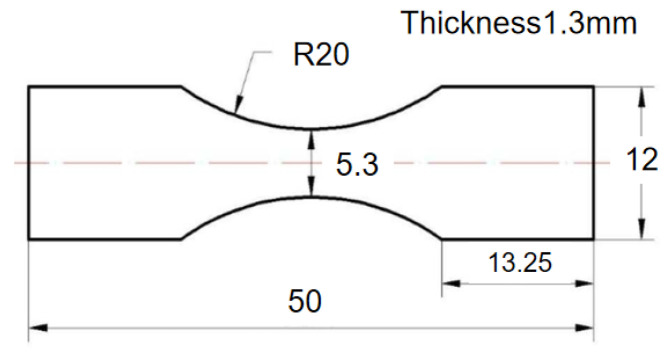
External dimensions of plate specimen (mm).

A traditional plate specimen is used as an experimental object, and its external dimensions are shown in Figure 3. The specimen surface is not finished, and the original surface state of the SLM process is retained. The CARE Measurement & Control M-5000 electromagnetic dynamic mechanical testing machine completes the high-cycle fatigue experiments. The loading frequency of the fatigue experiment is fixed at 30 hz (sinusoidal waveform) with a constant load ratio of R = 0.1, and the investigation is performed in the elastic area. Three specimens are tested at each stress level. If the difference between the failure cycles of the tested samples is greater than 40% of their average value, the 4th specimen is tested.

## 3. Results and Discussion

### 3.1. SLM Parameter Optimization for Cantor HEA

#### Effect of Energy Density on Part Density

**Figure 4:**
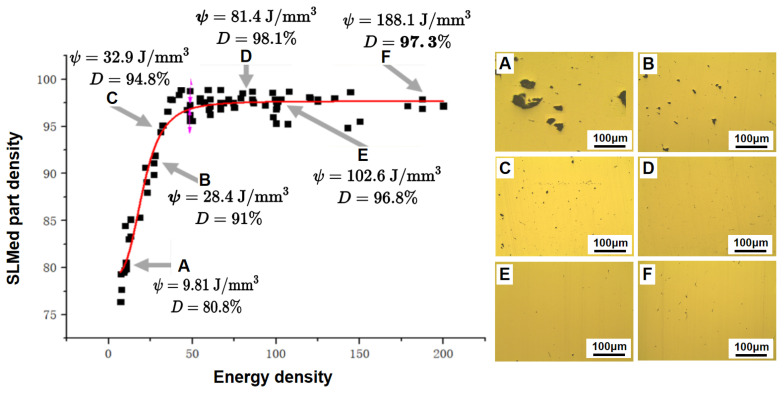
Relationship between energy density and SLMed part density.

To better analyze the quality of SLMed parts, an essential indicator for judging the properties of parts is introduced, namely density. The processing parameters (such as laser power, scanning speed, and scanning strategy) directly determine the density of the parts. The samples were built in a wide range of laser powers and scanning speeds in this work. The corresponding volumetric energy density is calculated by Equation (1), and the relationship between relative density and laser energy density is shown in Figure 4. By fitting the scattered points, the equation for the relative density D and laser energy density can be obtained:

(2) $$D=97.6817-\frac{18.5325}{1+\left(\frac{\psi }{20.6011}\right)}$$

As shown in Figure 4, when the volumetric energy density is less than 50 J/mm^3^ and other process parameters are constant, the density of the formed components significantly increases with increasing laser energy density, which is consistent with the results of other studies [42]. When the laser energy density is 66.7 J/mm^3^, the density of the parts is the highest, up to 98.87%. When the laser energy density further increases, the density of the parts decreases slightly and is finally kept stable at about 97%.

**Figure 5:**
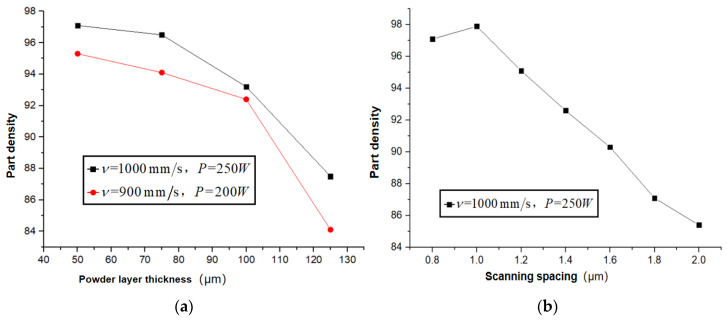
Effect of powder layer thickness (**a**) and scanning spacing (**b**) on SLMed part density.

Figure 5a illustrates the effect of powder layer thickness on part density. When the other machining parameters are constant, the part density increases with decreasing layer thickness. The upward trend becomes more pronounced at a small layer thickness because a large amount of air exists in the loose powder layer, which tends to be trapped as bubbles when the metal powder melts [43]. When other parameters are constant, the smaller the layer thickness, the deeper the laser beam can penetrate the layer, resulting in severer melting. At the bottom of the layer, a more significant number of bubbles move up to the surface of the layer and break up as the liquid solidifies rapidly, resulting in less porosity. Figure 5b shows the effect of the scanning spacing on part density. The part density exceeds 95% at a scanning spacing of 0.08–0.12 mm, because the minimum diameter of the laser spot is 0.1 mm. At least 20% of the overlap rate of the tracks can be guaranteed at a scanning spacing of 0.08–0.12 mm, and the density decreases from 97% to about 85% when the scanning spacing increases from 0.12 mm to 0.2 mm.

### 3.2. Analysis of Pore Defects in SLMed Parts

#### 3.2.1. Formation of Pore Defects

**Figure 6:**
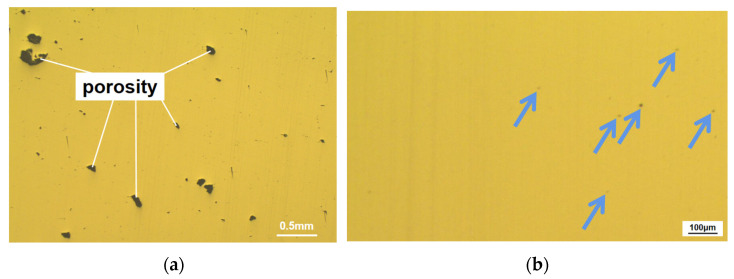
Cross-section of SLMed HEA sample with obvious (**a**) and permissible defects (**b**).

In Figure 6, many defects directly affect the part density. Specifically, the defects in SLMed parts are mainly classified into porosity and pore, in which the porosities are generally caused by a lack of fusion with a more complex geometry, as shown in Figure 6a. In contrast, pores are generally small and round, as shown in Figure 6b.

**Figure 7:**
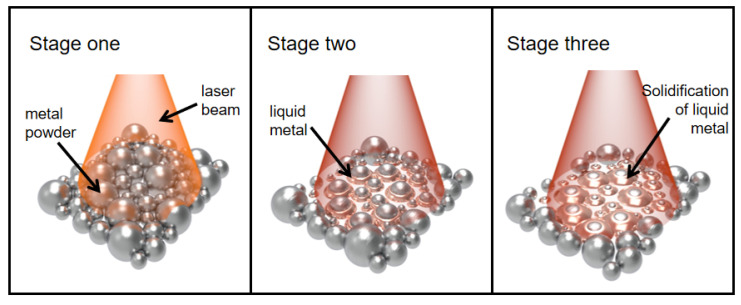
Schematic diagram of the pore formation mechanism.

**Figure 8:**
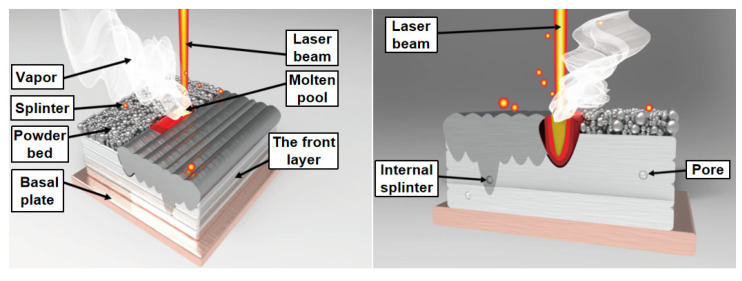
Defects in the SLM process.

1. When the laser beam scans through loose powder, the laser irradiates several powder particles simultaneously (Stage I), heating the particles. Subsequently, heat flows to the center of the particle until the particle temperature is locally steady [44]. Only the surface of the powder particles melts into a liquid at low laser energy, while the center remains solid. The liquid rapidly gathers between the particles, forming a liquid “bridge” (Stage II). When the laser is moved away, the liquid solidifies, and the surface-molten particles bind together, thus forming pores between the solidified metal agglomerate (Stage III). Figure 7 illustrates the pore formation mechanism.
2. Under laser irradiation, the melt in the molten pool inevitably splashes to either side of the molten pool, forming spherical metal or ablating into the forming surface and forming tiny pores. Some pores are formed during subsequent powder spreading, melting, and solidification, as shown in Figure 8.
3. The pores are mainly caused by moisture or contaminants on the surface of the powder particles [45], such as oxides.
4. Small amounts of oxygen and carbon in HEAs can react at high temperatures to form gaseous products, such as CO or CO_2_. Pores are included in the parts under the gas retention effect.
5. In addition, the particle size and the shape of powders are critical in reducing the number of defects in built-up layers [46,47].

#### 3.2.2. Effect of Energy Density on Pore Formation

Figure 4 shows the effect of laser density on part density. In the previous simulation, it was found that at low laser energy, the size of the molten pool is small, and the penetration depth is shallow. The metal powder can absorb less energy, not enough for complete melting, so the layers cannot be fully bonded, and some micro-bonding exists. Therefore, the viscosity of the melt is high, and the liquid phase is less mobile, resulting in a higher porosity of the parts.

The laser energy increases with increasing laser power. On the one hand, as the metal powder can absorb more energy, more powder particles are melted, and the amount of generated liquid is increased accordingly, even if the solid powder is not melted, thus improving the overall mobility, filling the pores and reducing the porosity with the help of liquid “bridge”. On the other hand, high energy directly heats the molten pool. The liquid metal more quickly flows at a relatively high temperature, which can fill the pores generated during the manufacturing process. The part density reaches the maximum, up to 98.87%, when the laser line energy density is increased to 66.7 J/mm^3^. In this case, most pores of the parts are round (diameter ≤ 10 μm), as shown in Figure 6b. As the laser energy rises, pore formation is no longer mainly caused by insufficient energy, but because the metal powder will become liquefied gas and form tiny pores under humidity. Moreover, the literature [48] argues that solidification shrinkage also leads to pore formation.

The part density slightly decreases with a further increase in the laser energy density. Scholars have discovered similar phenomena in other materials [37]. Many scholars have studied this phenomenon and believe that this phenomenon is mainly caused by the following: ➀ excessive laser energy density may lead to the elements at some low melting points evaporating, leaving some pores. ➁ Excessive laser energy density, and incredibly too slow scanning speed, bring about larger grains and more irregular and larger melt pools, which dramatically slows down the liquid solidification, thus triggering the balling and rupture in the molten pool, significant aggregation of particles in the liquid phase and formation of pores. ➂ A large molten pool is formed under high laser energy and is more susceptible to solidification micro-shrinkage [45]. ➃ At excessive laser power, if the scanning speed remains high, the “balling” phenomenon may occur in the manufacturing. The balling of a layer will bring about many irregular pores, directly leading to larger porosity of the parts [49]. In this case, we should increase the laser powder and reduce the scanning speed to manufacture a denser part. After the powder melts, a longer presentation time is available, facilitating the liquid flow, and thus manufacturing denser parts.

As the laser energy density further increases, especially when the laser power increases and the scanning speed decreases, the number of liquid phases increases, which generates a higher density. However, the density cannot keep increasing and tends to be stable at about 97%.

### 3.3. Microscale Mechanical Mechanism of Cantor HEAs

#### 3.3.1. Experiment on Micro-Scale Quasi-Static Compression Mechanics

**Figure 9:**
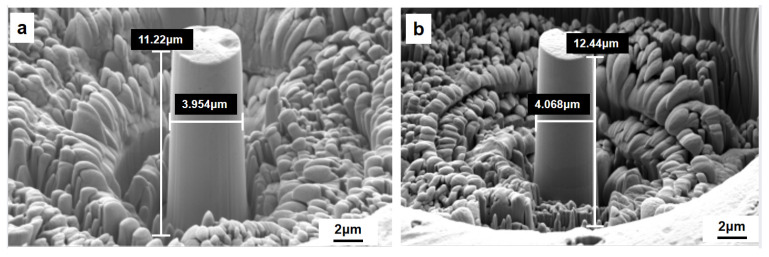
SEM image of FIB cone structure of SLM HEA: (**a**) FIB sampling section is perpendicular to the BD and (**b**) FIB sampling section is parallel to the BD.

**Figure 10:**
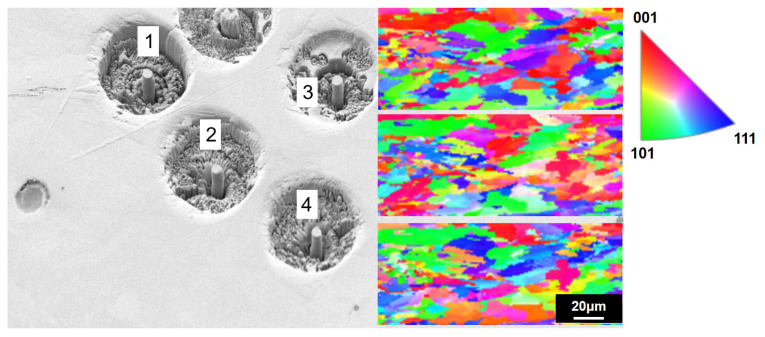
Micro-area SEM and IPF images of SLM HEA FIB cone structure sampling (note: the FIB sampling section is parallel to BD and FIB sampling as random polycrystalline microdomains).

An SEM in situ mechanical experimental device is used in this research to prepare a micro-scale cone structure from SLMed Cantor HEAs through FIB, as shown in Figure 9, which supports the in situ observation of the quasi-static compression behavior at the micro-scale level and the analysis of quantitative stress–strain data. The above FIB sample preparation corresponds to the inverse pole figure (IPF) for microstructure electron backscatter diffraction (EBSD), as shown in Figure 10, aiming to precisely determine the correspondence between the sampling location and the grain, as well as grain boundaries.

**Figure 11:**
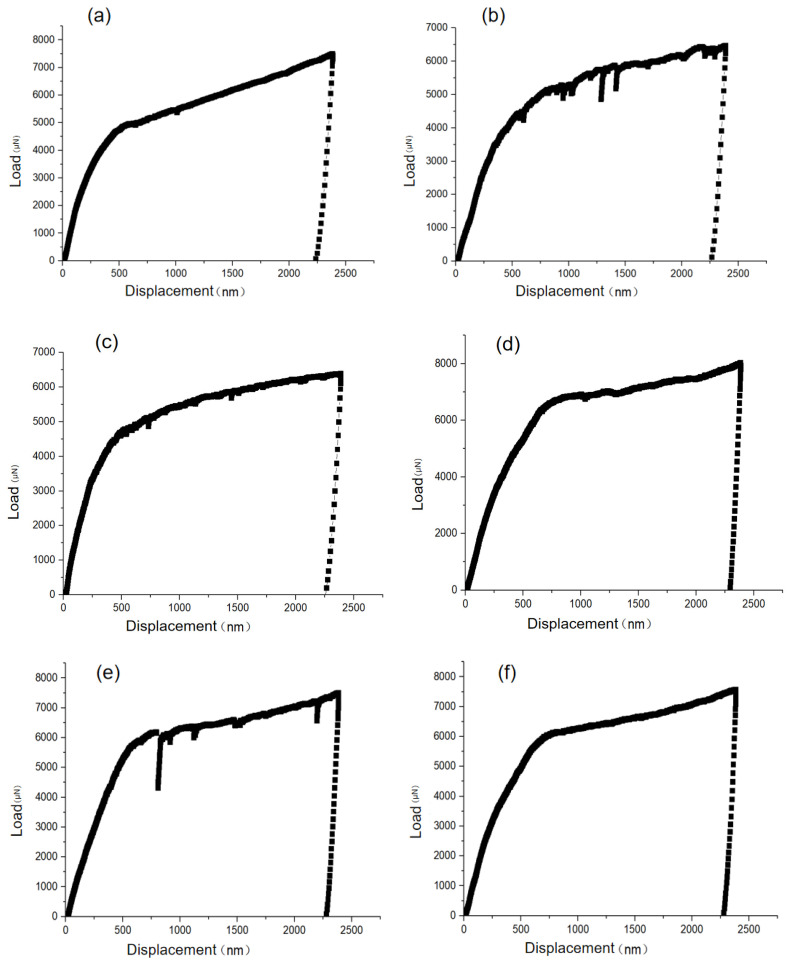
Typical sample micro-scale quasi-static compression load–displacement curve: (**a**–**c**) indicate the TD; (**d**–**f**) indicate the BD.

Figure 11 gives the micro-scale quasi-static compression load–displacement curves. Whether along the BD of the building orientation or the TD perpendicular to the building orientation, during the loading process of the FIB micro-scale cone with a diameter of about 3 μm~5 μm and a height of about 10 μm, small-scale stress relaxation and “dislocation avalanche” phenomenon repeatedly appear. However, in the overall loading process, there is a general trend of strain increase in the first half and failure in the second half. It should be noted that although the BD and TD directions of SLM forming present anisotropic characteristics at the structure level, with certain differences in grain morphology, orientation distribution, and mechanical properties. The mechanical properties are not anisotropic at the micro-scale level. Still, they are only related to the phase, composition, lattice structure properties, grain boundary density, and orientation in the FIB sampling volume range. What can be seen is that the SLMed HEAs have better overall micro-scale mechanical properties than the 316 L austenitic stainless steel with the same major components but significantly different atomic molar ratios due to its intrinsic properties of high-entropy effect and high lattice distortion, while the Cantor HEAs have a better corrosion resistance property, which points out a direction to following engineering material selection for additive manufacturing in same working conditions.

#### 3.3.2. Analysis of Micro-Scale Strength Mechanism

The SLMed CoCrFeNiMn HEA is organized as polycrystals, and crystal atoms are arranged in order. When a load is applied to a micro-structure of these crystal structures, the crystals will experience a micro-scale slip, inherent dislocations, and new dislocations and move under stress, resulting in excessive dislocations entangled within the metal and a local strengthening effect. When the applied stress further breaks through the yield stress threshold for local dislocation strengthening, the typical “dislocation avalanche” phenomenon occurs, driving the sudden movement of the entangled dislocations, thus weakening the metal strength, which is more difficult to control and a direct physical effect on the overall macroscopic failure behavior of the materials. The SEM micro-scale stress–strain experiments under in situ observation can accurately determine the location of the “avalanche” and its relationship with the loading direction and grain orientation.

Each metal atom has nuclei and electron clouds of different sizes, resulting in high distortion of the HEA lattice, which can theoretically slow down or even delay the motion of the entangled dislocations and thus store more dislocations in a relatively small volume. However, unlike traditional alloys, the HEA lattice and micro-structure accumulate more dislocation density, which also leads to more obvious “mutation” characteristics of stress and strain resulting from the “dislocation avalanche”, obviously detrimental to the sudden control of material failure.

**Figure 12:**
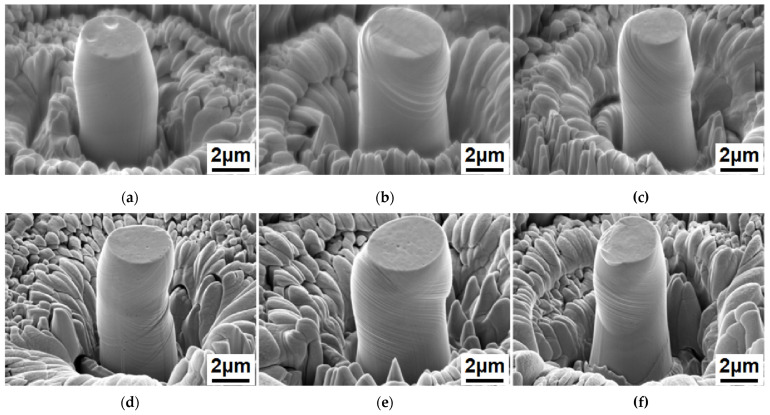
SEM morphology of SLM HEA FIB cone structure after in situ quasi-static compression: (**a**–**c**) FIB sampling section is perpendicular to the BD and (**d**–**f**) the FIB sampling section is parallel to the BD.

Figure 12 shows the SEM morphology of the FIB cone structure of SLMed CoCrFeNiMn HEA in BD and TD before and after quasi-static compression loading. It can be observed that significant non-uniform plastic deformation and slip bands appear in the microcolumn with increasing strain. By observing the contrast of the SEM microcolumn surface, we can find the local grain boundary of the polycrystal inside the micro-column. The slip band orientation shows noticeable differences on both sides of the grain boundary. The interlacing phenomenon of multiple groups of slip bands is observed in some samples. A new high-density dislocation is entangled in the interlaced area, thereby generating a unique strengthening effect.

#### 3.3.3. Effect of Forming Process on Micro-Scale Intrinsic Strength

The mechanical properties of macroscopic materials and microcolumns are significantly different in dislocation motion and strengthening mechanisms due to the size effect. From the viewpoint of micro-scale mechanics, the FIB sampling location and SLM process defects directly affect the microcolumn strength and damage resistance. For microcolumn sampling in this experiment, the SLM porosity and sparse process defects are avoided, as shown in Figure 10, thus avoiding the adverse effects of large defects on the mechanics of the samples.

**Figure 13:**
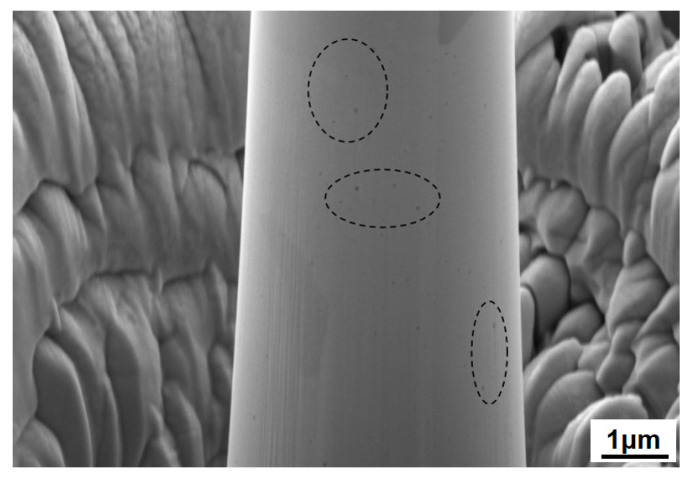
Nano-scale micro-pore clusters on the outer surface of SLM HEA FIB cone structure.

The investigation of the intrinsic strength of SLMed HEAs should exclude material discontinuities such as process defects. More focus should be put on the critical role of composition, crystal structure, grain orientation, and grain/subgrain boundaries. However, SLM forming process control plays a crucial role in improving the mechanical properties and service life of macroscopic parts. It has a certain effect on the mechanical properties of submicron and nano-scale materials. For example, as shown in Figure 13, nano-scale pores still exist in FIB microcolumn samples characterized by random dispersion. Such pores exist in a high-temperature molten pool under laser spot pressure as the result of open setting characteristics and environmental atmosphere of the powder bed during rapid laser-induced melting and solidification, and residue in solidified micro-structures because the solidification speed is faster than the rate at which pores float up and escape in a molten pool. It can be inferred that such nano-scale pores will still have a certain effect on the material mechanics, such as dislocation movement and slippage. When nanopores with a discontinuous atomic arrangement are encountered during dislocation movement, this type of defect shall be bypassed or cut, or new dislocation is entangled near the nanopores. Therefore, although the intrinsic strength of SLMed materials at the microscopic scale is related to the inherent structure, optimized process parameters for full-dense parts are the basis and prerequisite for improving the mechanical properties of the parts and are one of the critical factors to be considered in investigating microscale mechanics.

### 3.4. Research on High-Cycle Fatigue Properties of SLMed HEAs

#### 3.4.1. High-Cycle Fatigue Experiment Results

**Figure 14:**
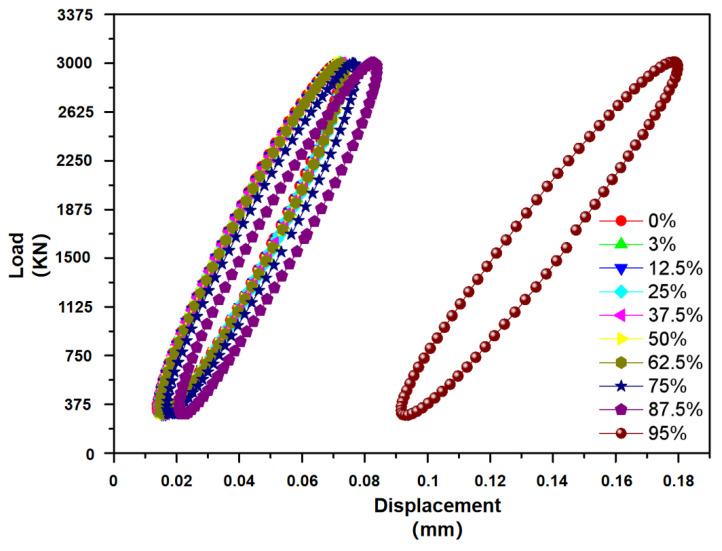
Load–displacement curve of high cycle fatigue.

**Figure 15:**
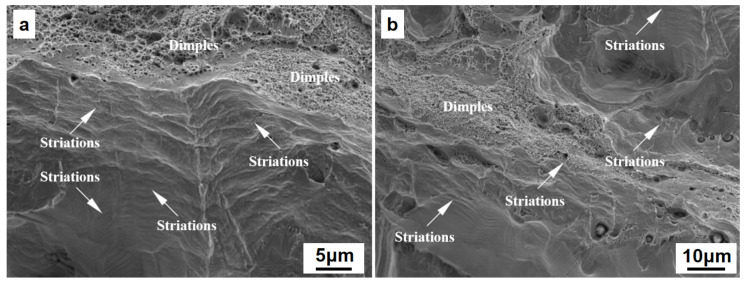
SEM image of typical SLM HEA high-cycle fatigue fracture: (**a**) loading direction is perpendicular to BD; (**b**) loading direction is parallel to BD (note: the arrow indicates the morphology of typical fatigue striations).

Figure 14 gives a high-cycle fatigue load–displacement curve of a typical specimen under tensile fatigue with a maximum stress of 200 MPa. During the experiment, the sample shows excellent cycle stability, and the hysteretic stress–strain curves are regular and sleek. With the increase in the number of cycles, the hysteretic curve shifts to right, indicating that the sample exhibits a slight ratcheting behavior under high-cycle fatigue loads. In addition, the area of hysteretic curves is basically unchanged, indicating that the sample does not have obvious cycle softening and hardening characteristics. At the late stage of fatigue life, there is a rapid translational motion for the hysteretic curve, which shows that the fatigue cracks have been formed in the specimen. Figure 15 shows typical fatigue striations on the fatigue failure fracture, and ductile and brittle fracture features such as dimples and cleavage steps are observed, indicating that the final fracture is a mixed-mode fracture. The observed fatigue striations reflect the propagation behavior of micro-cracks in the high-cycle and low-load cyclic loading process of the specimens. It should also be noted that a small number of process defects, such as incompletely melted powder clusters, porosity, or sparseness, are found in the sample fractures. Fatigue cracks are easily initiated and developed at these defects. Therefore, the increase in density is still the prerequisite for improving the mechanical fatigue properties and the service life under low-load alternating stress conditions.

#### 3.4.2. High-Cycle Fatigue Failure Mechanism and Micro-Analysis

**Figure 16:**
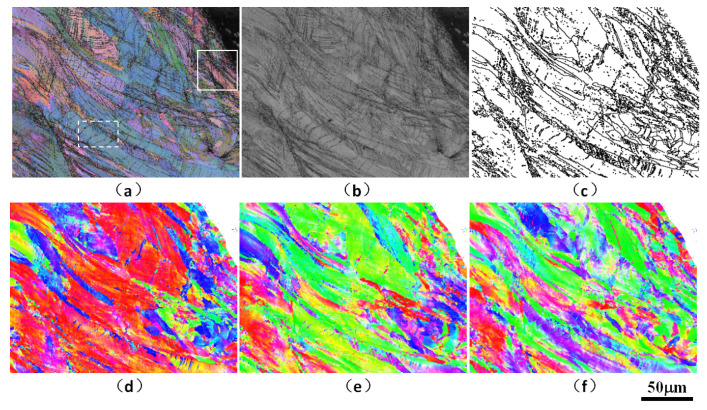
EBSD analysis results of microstructure near the fracture surface of the fatigue failure specimen of the SLM HEA: (**a**) comparison of all Euler diagrams and diffraction bands, (**b**) diffraction band comparison chart, (**c**) large angle (>15° black line) grain boundary diagram, (**d**) IPF_X inverse pole figure, (**e**) IPF_Y inverse pole figure, (**f**) IPF_Z inverse pole figure.

**Figure 17:**
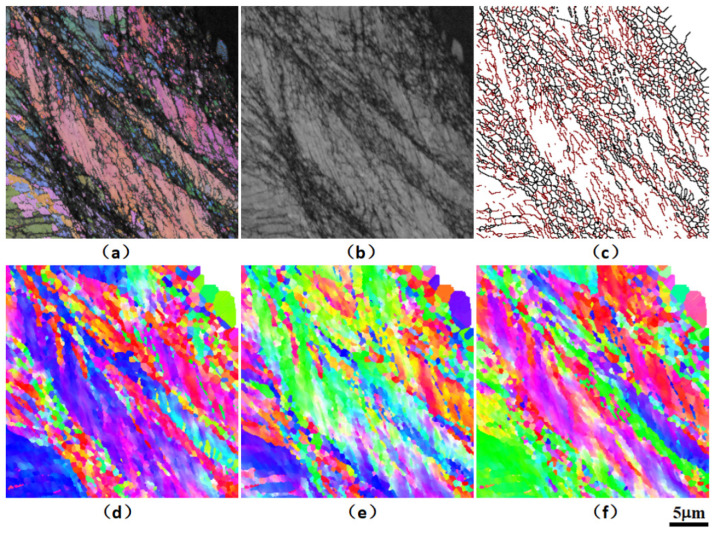
EBSD analysis results of microstructure near the fracture surface of the fatigue failure specimen of the SLM HEA (the detection area is the white solid frame area in Figure 16a): p.(**a**) comparison of all Euler diagrams and diffraction bands, (**b**) diffraction band comparison chart, (**c**) large angle (>15° black line) and small angle (<15° and >5° red line) grain boundary diagram, (**d**) IPF_X inverse pole figure, (**e**) IPF_Y inverse pole figure, (**f**) IPF_Z inverse pole figure.

**Figure 18:**
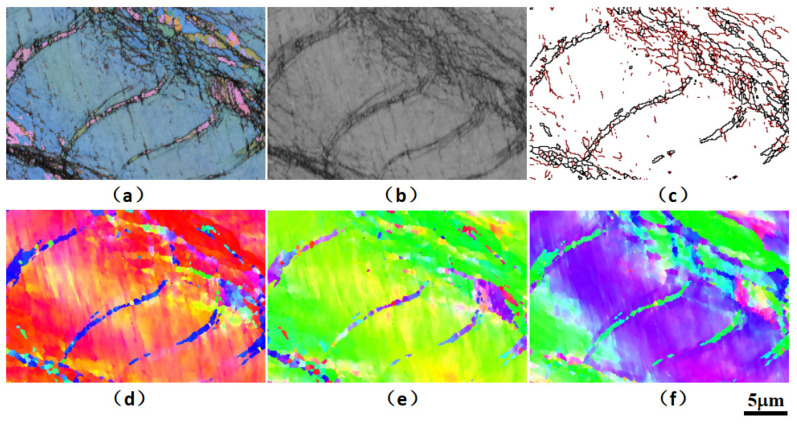
EBSD analysis results of microstructure near the fracture surface of the fatigue failure specimen of the SLM HEA (the detection area is the white dotted frame area in Figure 16a): p.(**a**) comparison of all Euler diagrams and diffraction bands, (**b**) diffraction band comparison chart, (**c**) large angle (>15° black line) and small angle (<15° and >5° red line) grain boundary diagram, (**d**) IPF_X inverse pole figure, (**e**) IPF_Y inverse pole figure, (**f**) IPF_Z inverse pole figure.

Aiming to further clarify the material micro-mechanism for different cyclic softening in the high-cycle fatigue process of SLMed parts, the fatigue failure samples are analyzed by micro-area EBSD, and the sampling area is close to the fracture or crack area. As shown in Figure 16, Figure 17 and Figure 18, the EBSD test reveals high-density nano-twin structure characteristics in the microstructure near the fatigue section.

The average grain size of SLM laser rapid solidification is significantly smaller than that of traditional quasi-static casting. At the same time, the intercrystalline substructure in the SLM microstructure and the aggregation of nano-oxide particles and heavy metal elements on the sub-micron and even nano-scale cell walls have a significantly higher dislocation pinning strengthening effect. More importantly, a high proportion of low-angle grain boundaries efficiently induces nano-twin nucleation under stress, and a large number of twin structures further enhance the plasticity and damage tolerance under structural strain, i.e., twin-induced plasticity (TWIP) mechanism, thus making a significant contribution to improving the plastic toughness synergy of the overall fine-crystalline SLMed parts. This can increase the strength of laser rapid non-equilibrium melting and solidification structure and play a positive role in prolonging low-load high-cycle fatigue lifetime in addition to certain or even better ductility.

#### 3.4.3. Effect of Process Parameters on Fatigue Properties of Cantor HEAs

As the laser energy density further increases, especially when the laser power increases but the scanning speed decreases, the number of liquid phases increases, producing a higher density. However, the density cannot keep increasing and tends to stabilize at 97% or so. During the SLM process, the pre-solidified parts and micro-structures are highly susceptible to accumulated residual stresses. Internal structural defects, including microcracks, balling, and porosity, generally occur under unacceptable process conditions, which significantly affect the mechanical fatigue properties of macroscopic parts.

For example, uneven structure thermal stress releasing caused by laser rapid non-equilibrium melting and solidification, uneven solid–liquid interface solidification, and remarkable shrinkage effect resulting from incomplete melting of local powder as well as mismatching laser parameters with the set thickness of powder layer are factors easily leading to buckling deformation and the generation of macro cracks in parts, or the generation of micro-cracks inside the part. Additionally, balling evoked by inappropriate laser parameters is a common defect that exerts a major effect on the quality and mechanical properties of the parts in the SLMed parts or on the SLM surface. Meanwhile, pores are frequently found in layer-by-layer laser scanning due to balling defects independent of morphological characteristics. Consequently, excessive porosity declined density, and unsatisfactory surface roughness is caused in the parts. What is more, incompletely melted local powder results from remaining pores in the molten pool, excessive laser scanning distance or powder layer thickness will also give rise to many pore defects. In particular, if the gas phase is introduced into the molten pool via the rolled winding or the laser spot pressure fails to spill before solidification in the rapid solidification of the molten pool, it will easily be remained in the solid structure, forming tiny spherical pores. The remaining pore defect is a common phenomenon in the laser “keyhole” effect. The remaining locations of the process defects in the core of the parts or the outer surface with greater roughness are potential areas susceptible to fatigue crack, which is the main cause of premature failure of the parts under fatigue load. Branch cracks in the initiation and development process are straightforward to develop again into the main crack along the defective parts and bring severer damage. Existing studies have found that core defects are the main cause of fatigue failure in terms of high-cycle fatigue of the SLM metal parts, i.e., fatigue cracks are only initiated and expanded from the core, highly different from the case where high-cycle fatigue is preferentially initiated and expanded on the surface compared with conventional forgings made of ideal materials. It should be noted that numerous studies have demonstrated that the density of the parts is increased by optimizing the SLM process parameters. The risk of premature fatigue failure of the parts is reduced through appropriate post-treatment processes, especially HIP post-treatment for entire parts, and mechanical densification/fine crystallization post-treatment for the surface or surface layer of the AM parts can significantly improve fatigue performance.

It should be noted that the effect of SLM additive manufacturing microstructure anisotropy on fatigue performance is also considered. Various metal structures suitable for SLM manufacturing are comparable to their casting and forging materials in quasi-static strength. Still, their fracture toughness is generally low, and the discrete characteristics of fatigue performance test data are significant, which is not only related to process defects but also significantly related to the characteristics of the SLM microstructure. For metallic materials with a single matrix phase or main phase, the effect mechanism of grain size and its distribution characteristics, grain boundary angle distribution characteristics, grain morphology and texture, and intercrystalline substructure (especially intercrystalline cellular substructure unique to the SLM microstructure made of several common metallic materials) of SLM part micro-structures on the mechanical fatigue performance has attracted the attention of relevant researchers. The SLMed parts present microstructural texture characteristics, and growth characteristics of columnar grains lead to significant anisotropy in mechanical properties, which directly affects the fracture behavior or the preferred orientation of crack growth; in practice, by changing the laser heat source parameters, the grain orientation can be controlled in some way to improve the anisotropy of mechanical properties, for example, higher laser energy density leads to preferential growth of FCC grains along <001>. In comparison, lower energy density leads to preferential growth of grains along <110>. The <110> grain orientation facilitates the formation of a large number of twin crystals in the austenite structure in case of deformation. It results in a higher strain hardening rate, increasing the strength limit and ductility of the structure. However, in general cases, the crystalline structure also exhibits unavoidable differences in orientation characteristics along the SLM building direction.

## 4. Conclusions

Given SLM process characteristics, this research investigates the process window of SLM forming of Cantor alloy, analyzes the effect law of different process parameters on the forming quality, microstructure, and mechanical properties of Cantor HEAs, and draws the following conclusions:

1. The SLM parameter is optimized based on density. The results show that the increase in laser energy density can significantly improve the quality of the parts when other process parameters remain unchanged. The density of the parts can reach up to 98.87%, but the density of the parts will slightly decrease with increasing laser energy, and finally tend to stabilize. The part density decreases with increasing layer thickness and scanning spacing.
2. The causes of porosity defects and the effect of process parameters are elaborated. Pore defects are caused by metal agglomeration, spatter, moisture or contaminants, gaseous products, etc.; the laser energy increases with increasing temperature and fluidity of the molten pool, in which case pore defects are easily eliminated, but the pores in the final parts cannot be eliminated due to moisture, solidification shrinkage, etc.
3. This research analyzes the effect of different typical SLM process conditions on the quasi-static and dynamic mechanical properties, investigates the strength and toughness of the micro-scale and macro-scale samples, reveals the stress–strain changes resulting from repeated slippage in quasi-static compression behavior of micro-scale materials and “dislocation avalanche” behavior mechanism, and finds that laser rapid non-equilibrium melting and solidification lead to that a large number of nano twin crystals induced by fatigue stress exist near high-density low-angle grain boundaries and micro-plastic deformation and ductility are improved in the low-load high-cycle fatigue process.

## Figures and Tables

**Table 1:** Primary working parameters of the SLM platform.

Parameter	Setting
Rated output power/W	≥500
Center wavelength/nm	1060–1080
Output power fluctuation	≤3%
Minimum spot diameter/mm	≤0.1

## Data Availability

Data available in a publicly accessible repository.
